# Overdose, opioid treatment admissions and prescription opioid pain reliever relationships: United States, 2010–2019

**DOI:** 10.3389/fpain.2022.884674

**Published:** 2022-08-04

**Authors:** Larry Aubry, B. Thomas Carr

**Affiliations:** ^1^Independent Researcher, Hampshire, IL, United States; ^2^Carr Consulting, Wilmette, IL, United States

**Keywords:** prescription opioid sales, overdose deaths, opioid treatment admissions, CDC guideline, statistical analysis, analysis of historical data

## Abstract

**Background:**

“As part of the U.S. government's urgent response to the epidemic of overdose deaths (1)” the United States Centers for Disease Control and Prevention (CDC) issued the “CDC Guideline for Prescribing Opioids for Chronic Pain-United States, 2016 (2)” (guideline) followed by the “CDC Clinical Practice Guideline for Prescribing Opioids–United States, 2022 (3) (guideline update). ” The guideline and guideline update cite a direct correlation between prescription opioids sales (POS) and opioid treatment admissions (OTA) and prescription opioid deaths (POD), which was based on data from 1999 to 2010. This paper updates those relationships and includes the correlations between prescription opioid sales (POS) and any opioid deaths (AOD) and total overdose deaths (TOD) from 2010 to 2019.

**Methods:**

Linear regression models were fit to each response separately. Opioid sales (measured as MME (morphine milligram equivalent) per capita) was the independent variable. Total overdose deaths (TOD), any opioid overdose deaths (AOD), prescription opioid overdose deaths (POD) and opioid treatment admissions (OTA) were the dependent, response variables. The models were assessed using three criteria: the statistical significance of the model (Overall *P*-Value), the quality of the fit (*R*^2^), and the sign of the slope coefficient (positive or negative).

**Results:**

The analyses revealed that the direct correlations (i.e., significant, positive slopes) reported by the CDC based on data from 1999 to 2010 no longer exist. Based on data from 2010 to 2019, the relationships either have reversed (i.e., significant, negative slopes) or are non-existent (i.e., no significant model).

**Conclusions:**

The guideline, guideline update, CDC's public, medical profession, and intergovernmental communications should be corrected/updated to state no direct correlation has existed between POS to OTA, POD, AOD, and TOD since 2010. Individualized patient care and public health policy should be amended accordingly.

## Background and rationale

**Figure 1:**
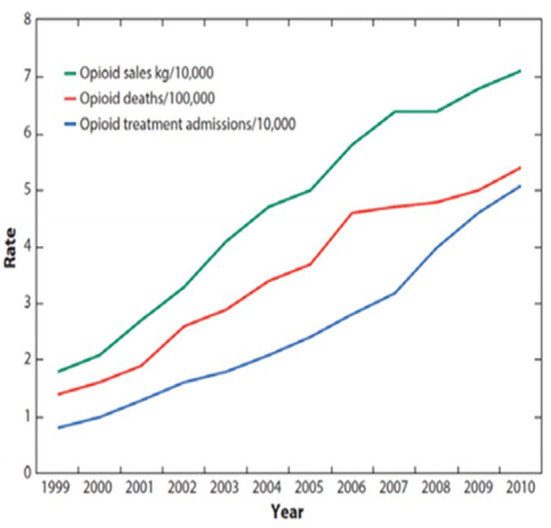
CDC chart 1999–2010, February 28, 2018, Congressional testimony “Combatting the Opioid Crisis,” made before the Committee on Energy and Commerce, Subcommittee on Health U.S. House of Representatives (5): “The CDC has shown that a sharp increase in prescriptions for opioids resulted in a corresponding rise in addiction and overdose deaths. This is a CDC graph. The green line represents opioid prescribing, the red line represents opioid deaths, and the blue line represents opioid addiction. The green line went up as opioid prescriptions started to soar, it led to parallel increases in addiction and overdose deaths (6)”.

Direct correlations that existed between Prescription Opioid Deaths (POD), Opioid Treatment Admissions/addiction (OTA) and Prescription Opioid Sales (POS) from 1999 to 2010 (4) (see Figure 1) led the CDC to conclude that POS are the determinant for POD, any opioid overdose deaths (AOD), Total overdose deaths (TOD), and OTA (1–14).

The U.S. Department of Health and Human Services (HHS) declared in 2015 “There is a clear correlation between opioid prescribing rates and overdose death rates in the United States (7).” With the guideline's release, then CDC Director Tom Frieden stated, “Overprescribing opioids—largely for chronic pain—is a key driver of America's drug-overdose epidemic (1).”

Cutting POS has been CDC's, DEA's, legislative policy makers', healthcare system providers and practitioners' solution to cut overdose deaths and OTA (1–18).

The impact of the CDC guideline has been systemic. Long term opioid therapy patients are not accepted as new patients by over 40% of primary pain clinics (18). 2021 MME per capita use declined to 309 (19), a level last seen in 2000, while the over 55 population with its age-related health conditions increased by 40 million since then and COVID care has required “high demand.” “Forty-seven states and the District of Columbia have laws that set time or dosage limits for controlled substances (20).” “All 50 states have established prescription drug monitoring programs (PDMPs) (21)” to collect and surveil doctor, patient, and dispensed medication information. Since 2009, U.S. morphine milligram equivalents per 1,000 inhabitants per day (MID) declined by 48% from second in the world (22) to third in 2019 (23).

The American Medical Association (AMA) reports “72% of pain medicine specialists said that they—or their patients—have been required to reduce the quantity or dose of medication they have prescribed (24)” as a result of the guideline.

The objective of the guideline was to cut opioid addiction and overdose deaths while ensuring to first do no harm. Considering “The epidemic of overdose deaths in the USA has been growing, inexorably and exponentially, for four decades” per the U.S. Drug Enforcement Administration (DEA) (25), an increase in U.S. overdose deaths of nearly 70% from 2016 to 2021, and an annual overdose cost of $1 trillion in the United States (26),” it is critical that public health policy and individual patient care not be based on out-of-date or misleading information.

The 2022 guideline update revises and expands upon the recommendations of the 2016 guideline considering a substantial amount of more recent data. However, it continues to cite the positive relationship between opioid prescribing rates and overdose deaths between 1999 and 2010 but makes no mention of the fact that those relationships have not existed for more than a decade. It is important that both clinical practice and regulatory policy be based on as much valid data as is readily available. This paper is intended to augment the new information contained in the guideline update to address the current relationships between POS and OTA, POD, AOD, and TOD.

The direct correlation of POS with OTA, POD, AOD and TOD has been cited in communications of public health policy, individual patient care and doctor conduct by HHS and CDC, referenced in congressional testimony, intergovernmental communications, and legal proceedings, thereby making these correlations a critical material fact. The analyses presented in this paper covering the period from 2010 to 2019 updates these material facts to avoid misrepresentation or omission of relevant evidence.

## Methods

### Description of data sources

#### Data limitations

Data limitations have the potential for over or underestimating overdose deaths. The authors of a 2018 report “Quantifying the Epidemic of Prescription Opioid Overdose Deaths,” with the CDC, acknowledged that systemic errors and omissions in the source data along with the CDC's methodology for compiling drug-related mortality data “could significantly inflate (27)” prescription opioid overdose death estimates (27, 28). In 2018, the CDC cut their estimates of prescription opioid deaths from 1999 to 2016 by 48,000 or 19.5%, with the 2016 estimates cut by more than 15,000 or 47.3% (27, 28).

Confounding factors impacting the accuracy of overdose deaths are that “multiple drugs are often involved” (27), the source of opioids detected in postmortem blood toxicity screens is not known (e.g., legally prescribed vs. illicitly obtained), among other issues (27, 28). With this occurrence and/or when multiple conditions resulted in an overdose death a single sequence/cause will be documented based on the physician's “best medical opinion (29).”

The same data sources that the CDC guideline appears to be based upon were used for this paper. As such, the results of analyses presented here are at least as reliable and subject to the same limitations as what the CDC obtained from their own analyses of 1999–2010 and if they chose to undertake them for the most recent decade of 2010–2019. Thus, the following sources have been applied.

**Drug Overdose Deaths (National); Total Overdose Deaths, Any Opioid Overdose Deaths and Prescription Opioid Overdose Deaths** (30): 1999–2019 data accessed from Drugabuse.gov., Published 2021. Deaths are classified according to the International Classification of Diseases, 10th Revision. Drug overdose deaths are identified with underlying cause-of-death codes X40–X44, X60–X64, X85, and Y10–Y14. The following multiple cause-of-death codes were used to identify specific drug types: T40.2 for natural and semisynthetic opioid analgesics, T40.3 for methadone, and T40.4 for synthetic opioid analgesics other than methadone. Accessed January 10, 2021 https://www.drugabuse.gov/sites/default/files/Overdose_data_1999-2019.xlsx.

**Opioid Overdose Death Crude Rates (U.S. States)** (31): 1999–2019 data accessed from CDC, National Center for Health Statistics. Underlying Cause of Death, 1999–2019 were sourced from CDC WONDER Online Database, released in 2020. Data are from the Multiple Cause of Death Files, 1999–2019, as compiled from data provided by 57 vital statistics jurisdictions through the Vital Statistics Cooperative Program. Identified using underlying cause-of-death codes X40–X44, X60–X64, X85, and Y10–Y14. Accessed Feb 7, 2021, 12:01:39 PM from http://wonder.cdc.gov/ucd-icd10.html.

**Opiate/Opioid Treatment Admissions (National)** (32): 2006–2008 data accessed from Substance Abuse and Mental Health Services Administration, Center for Behavioral Health Statistics and Quality, “Treatment Episode Data Set (TEDS): 2000–2010”. National Admissions to Substance Abuse Treatment Services. DASIS Series S-61, HHS Publication No. (SMA) 12-4701. Rockville, MD: Substance Abuse and Mental Health Services Administration (samhsa.gov), 2012. P. 43. Accessed April 18, 2021 from Treatment Episode Data Set (TEDS) *2000–2010* (samhsa.gov).

**Opiate/Opioid Treatment Admissions (National)** (33): 2008–2018 data accessed from Substance Abuse and Mental Health Services Administration, Center for Behavioral Health Statistics and Quality, “Treatment Episode Data Set (TEDS): 2018.” Admissions to and Discharges from Publicly Funded Substance Use Treatment. Rockville, MD: 2018 TEDS Annual Report. Substance Abuse and Mental Health Services Administration (samhsa.gov), 2020. Table 1.1a. Accessed April 18, 2021 from https://www.samhsa.gov/data/sites/default/files/reports/rpt31097/2018_TEDS/2018_TEDS.html#PSU. *2018* TEDS Annual Report (samhsa.gov).

**Opioid Prescribing; MME per Capita (National)** (34): 2006–2013 data accessed from CDC,” Annual Surveillance Report of Drug-Related Risks and Outcomes—United States Surveillance Special Report” 0.2019 CDC, U.S. Department of Health and Human Services. Published November 1, 2019. P. 115. Accessed January 10, 2021 from https://www.cdc.gov/drugoverdose/pdf/pubs/2019-cdc-drug-surveillance-report.pdf.

**Opioid Prescribing; MME per Capita (National**) (35): 2014–2018 data accessed from Statista,” Annual morphine milligram equivalents (MME) dispensed per capita in the U.S. from 2014 to 2018”, MME per capita U.S. 2014–2018. Statista. May 28, 2021. Accessed July 8, 2021 from https://www.statista.com/statistics/753284/number-of-mme-dispensed-per-capita-in-us/.

**Opioid Prescribing; MME per Capita (National**) (36): 2019 data accessed from The IQVIA Institute, “Prescription Opioid Trends in the United States,” Institute Report, Dec 16, 2020. P.4. Accessed January 10, 2021 from https://www.iqvia.com/insights/the-iqvia-institute/reports/prescription-opioid-trends-in-the-united-states.

**Opioid Prescribing; Opioid Dispensing Rates per 100 (U.S. States)** (37): 2006–2019 data accessed from CDC, “U.S. Opioid Dispensing Rate Maps, Drug Overdose,” CDC Injury Center. Accessed February 9, 2021 from https://www.cdc.gov/drugoverdose/rxrate-maps/index.html.

**Opioid Sales kg/10,000 (National):** For the period from 2006 through 2018/2019, these data were not known to be publicly available. We instead examined Opioid Prescribing by separately computing MME per Capita.

### Statistical methodology

#### Objective 1: Evaluate MME per capita as a legitimate alternative measure of annual prescription opioid sales

The CDC used Annual Prescription Opioid Sales to support the guideline (Figure 1). Data on Annual Prescription Opioid Sales are not readily available since 2010. However, MME per Capita data are available from 2006 to 2019 and offer a reasonable surrogate. Annual Sales data from the CDC chart were visually extracted and correlated with MME per Capita data, using simple linear regression analysis. The goal of the analysis was to evaluate MME per Capita as a legitimate alternative measure of Annual Prescription Opioid Sales.

#### Objective 2: Assess the strength and nature of the relationships between total overdose deaths, any opioid overdose deaths, prescription opioid overdose deaths and opioid treatment admissions and opioid sales/MME per capita

Consistent with the methods used by the CDC, simple linear regression models were fit to the data. Separate models were fit to each of the four dependent variables (TOD, AOD, POD, and OTA) using Annual Opioid Sales (i.e., MME per Capita) as the independent variable. Two models were fit to each dependent variable. One model covered the years presented in the original CDC chart (for which MME per Capita data were available) (2006–2010) and the second model covered the years since the published CDC chart (2010–2019).

For both objectives, the strength and nature of relationships in all the regression models were assessed using three criteria:

1) significance of the regression model (overall *P*-Value),

2) the quality of the model's fit (*R*^2^), and

3) the sign of the linear slope coefficient (+ or –).

All models were fit using PROC REG from SAS/STAT software Version 9.4.

## Results and discussion

Data from CDC's original chart was reconstructed using graphical analysis. The reconstructed Annual Prescription Opioid Sales values from the original CDC chart are highly correlated with publicly available MME per Capita values (*R*^2^ = 94%). MME per Capita, which are available for more recent years than the data originally used by the CDC, is thus used in place of Annual Prescription Opioid Sales for all subsequent analyses.

For the years covered in the CDC's original chart (for which MME per Capita data are available, i.e., 2006–2010), the CDC's claim of positive/direct relationships between TOD, AOD, POD, and OTA and Annual Prescription Opioid Sales (i.e., MME per Capita) were validated (91% < *R*^2^ <97%), with statistically significant, positive slopes.

**Figure 2:**
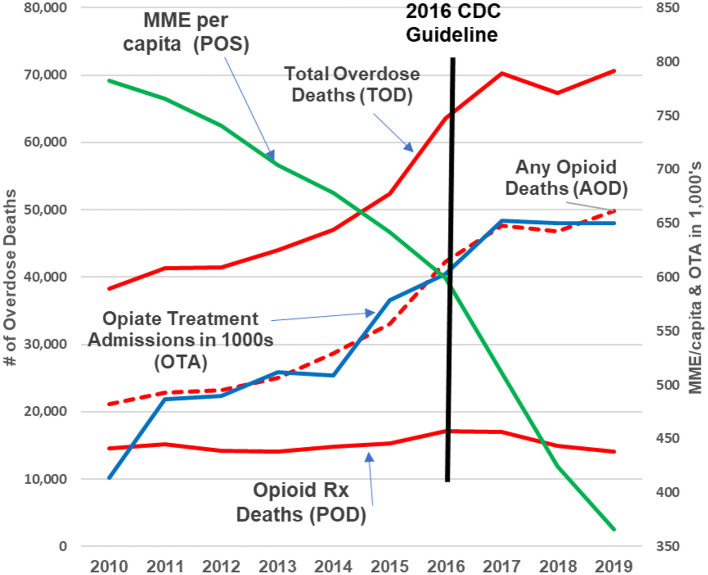
2010–2019 update. The green line represents opioid prescribing (POS, MME/capita); the red lines are opioid deaths (POD, AOD, and TOD); the blue line represents opioid addiction (OTA). Over the past decade, as the green line (prescription opioids) declined by +50%, prescription opioid deaths remained flat while opioid addiction, any opioid and total overdose deaths continued increasing “exponentially (9)”.

**Figure 3:**
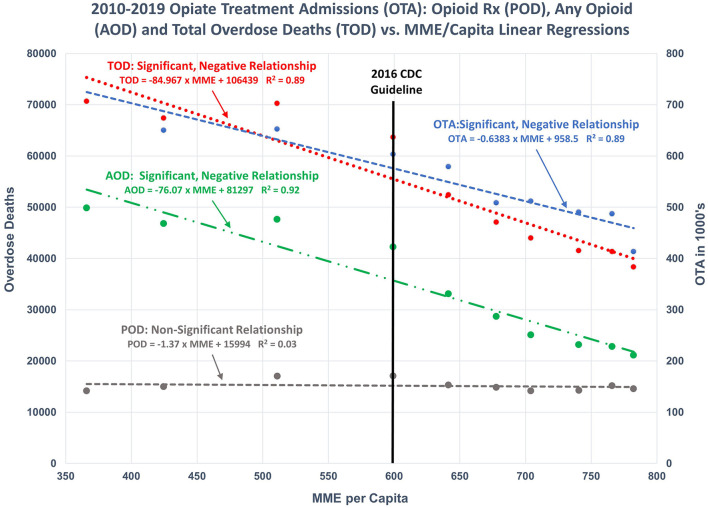
2010–2019 regression models: Illustrates the regression of OTA, POD, AOD, and TOD as functions of POS. Significant, negative relationships were found for OTA, AOD, and TOD. No significant relationship exists between POD and POS.

For more recent years (i.e., 2010–2019), however, the CDC's assertion of continued direct relationships is not valid. The relationships between TOD, AOD, POD, and OTA and Annual Prescription Opioid Sales (i.e., MME per Capita) are either non-existent or significantly negative/inverse (Figures 2, 3).

Results for all regression models are presented in Table 1.

**Table 1:** Summary of national regression models fit in the paper.

**Dependent variable**	**Independent variable**	**Years**	**Related figure**	***n***	***R* ^2^**	***P*-value**	**Slope**	**95% LCL**	**95%UCL**	**Interpretation**
MME per capita	Total annual prescription opioid sales	2006–2010	NA	5	0.94	0.006	141	76	206	Strong model. Significant, positive relationship.
Total overdose deaths	MME per capita	2006–2010	NA	5	0.97	0.002	20	14	26	Strong model. Significant, positive relationship.
Any opioid overdose deaths	MME per capita	2006–2010	NA	5	0.99	0.000	20	17	24	Strong model. Significant, positive relationship.
Prescription opioid deaths	MME per capita	2006–2010	NA	5	0.97	0.002	15	10	20	Strong model. Significant, positive relationship.
Opioid treatment admissions/1,000	MME per capita	2006–2010	NA	5	0.91	0.011	0.60	0.26	0.94	Strong model. Significant, positive relationship.
Total overdose deaths	MME per capita	2010–2019	Figure 3	10	0.89	0.000	−85	−109	−61	Strong model. Significant, negative relationship.
Any opioid overdose deaths	MME per capita	2010–2019	Figure 3	10	0.92	0.000	−76	−95	−57	Strong model. Significant, negative relationship.
Prescription opioid deaths	MME per capita	2010–2019	Figure 3	10	0.03	0.615	−1.4	−7.4	4.7	No Model. Nonsignificant relationship
Opioid treatment admissions/1,000	MME per capita	2010–2018	Figure 3	9	0.89	0.000	−0.64	−0.84	−0.43	Strong model. Significant, negative relationship.

National trends since 2010 are paralleled in a strong majority of states. Between 2010 and 2019 inclusive, there was a statistically significant negative correlation (95% confidence level) between AOD and Annual Prescription Opioid Sales in 38 states, with significant positive correlations occurring in only 2 states. Ten (5) states did not exhibit a significant (95% confidence level) relationships between overdose deaths and prescription opioid sales during the 2010–2019 time period (Table 2).

**Table 2:** Summary of regression models by state, any opioid overdose death by opioid prescribing rate/100 people.

**State**	**n**	**R^2^**	**P-Value**	**Slope**	**95% LCL**	**95% UCL**	**Interpretation**
AK	10	0.13	0.300	−0.07	−0.23	0.08	Nonsignificant relationship
AL	10	0.52	0.019	−0.08	−0.14	−0.02	Significant negative relationship
AR	10	0.22	0.174	−0.04	−0.11	0.02	Nonsignificant relationship
AZ	10	0.97	0.000	−0.19	−0.22	−0.17	Significant negative relationship
CA	10	0.84	0.000	−0.14	−0.19	−0.09	Significant negative relationship
CO	10	0.55	0.014	−0.09	−0.16	−0.02	Significant negative relationship
CT	10	0.92	0.000	−0.75	−0.93	−0.57	Significant negative relationship
DE	10	0.88	0.000	−0.36	−0.46	−0.25	Significant negative relationship
FL	10	0.54	0.015	−0.28	−0.48	−0.07	Significant negative relationship
GA	10	0.59	0.009	−0.09	−0.16	−0.03	Significant negative relationship
HI	10	0.86	0.000	−0.29	−0.39	−0.19	Significant negative relationship
IA	10	0.29	0.109	−0.06	−0.15	0.02	Nonsignificant relationship
ID	10	0.76	0.001	−0.11	−0.16	−0.06	Significant negative relationship
IL	10	0.84	0.000	−0.52	−0.70	−0.34	Significant negative relationship
IN	10	0.86	0.000	−0.25	−0.34	−0.17	Significant negative relationship
KS	10	0.52	0.019	−0.08	−0.14	−0.02	Significant negative relationship
KY	10	0.62	0.007	−0.15	−0.24	−0.05	Significant negative relationship
LA	10	0.88	0.000	−0.34	−0.44	−0.24	Significant negative relationship
MA	10	0.91	0.000	−0.68	−0.85	−0.51	Significant negative relationship
MD	10	0.92	0.000	−0.98	−1.22	−0.74	Significant negative relationship
ME	10	0.86	0.000	−0.40	−0.54	−0.27	Significant negative relationship
MI	10	0.73	0.002	−0.29	−0.43	−0.14	Significant negative relationship
MN	10	0.76	0.001	−0.17	−0.25	−0.09	Significant negative relationship
MO	10	0.92	0.000	−0.29	−0.36	−0.22	Significant negative relationship
MS	10	0.37	0.063	−0.03	−0.06	0.00	Nonsignificant relationship
MT	10	0.14	0.290	−0.03	−0.10	0.03	Nonsignificant relationship
NC	10	0.80	0.000	−0.27	−0.37	−0.16	Significant negative relationship
ND	10	0.71	0.002	−0.24	−0.37	−0.11	Significant negative relationship
NE	10	0.43	0.038	−0.09	−0.17	−0.01	Significant negative relationship
NH	10	0.55	0.014	−0.43	−0.75	−0.11	Significant negative relationship
NJ	10	0.93	0.000	−0.87	−1.06	−0.68	Significant negative relationship
NM	10	0.33	0.080	−0.09	−0.19	0.01	Nonsignificant relationship
NV	10	0.00	0.938	0.00	−0.06	0.05	Nonsignificant relationship
NY	10	0.81	0.000	−0.60	−0.83	−0.36	Significant negative relationship
OH	10	0.77	0.001	−0.45	−0.65	−0.25	Significant negative relationship
OK	10	0.45	0.035	0.04	0.00	0.08	Significant positive relationship
OR	10	0.04	0.604	−0.01	−0.05	0.03	Nonsignificant relationship
PA	10	0.70	0.002	−0.57	−0.88	−0.27	Significant negative relationship
RI	10	0.78	0.001	−0.31	−0.45	−0.18	Significant negative relationship
SC	10	0.91	0.000	−0.24	−0.30	−0.18	Significant negative relationship
SD	10	0.61	0.007	−0.25	−0.41	−0.09	Significant negative relationship
TN	10	0.96	0.000	−0.21	−0.24	−0.17	Significant negative relationship
TX	10	0.68	0.003	−0.04	−0.06	−0.02	Significant negative relationship
UT	10	0.04	0.570	−0.03	−0.17	0.10	Nonsignificant relationship
VA	10	0.80	0.000	−0.26	−0.36	−0.15	Significant negative relationship
VT	10	0.28	0.115	−0.51	−1.17	0.15	Nonsignificant relationship
WA	10	0.74	0.001	−0.06	−0.08	−0.03	Significant negative relationship
WI	10	0.82	0.000	−0.24	−0.33	−0.15	Significant negative relationship
WV	10	0.81	0.000	−0.28	−0.39	−0.17	Significant negative relationship
WY	10	0.56	0.013	0.13	0.04	0.23	Significant positive relationship

The guideline emphasized to clinicians that opioid dosages should be limited to no more than 90 MME/day based on the “evidence regarding the association of opioid dosage and overdose risk” in that “overdose risk is increased at higher opioid dosages” (2).

This recommendation is not supported by the available data. Regression analyses of TOD, AOD, and OTA on POS from 2010 to 2019 among patients receiving doses of at least 90 MME/day show significant negative relationships, indicating that lower POS in this high-dosage cohort do not correspond to lower death rates. As with the national results, the relationship between POD and POS in this cohort is not significant (Table 3).

**Table 3:** Summary of > 90 MME regression models.

**Dependent variable**	**Independent variable**	**Years**	***n***	***R* ^2^**	***P*-Value**	**Slope**	**95% LCL**	**95%UCL**	**Interpretation**
Total overdose deaths	Prescriptions/100 people	2010–2019	10	0.83	0.000	−4,677	−6,394	−2,961	Strong model. Significant, negative relationship.
Any opioid overdose deaths	Prescriptions/100 people	2010–2019	10	0.84	0.000	−4,157	−5,623	−2,692	Strong model. Significant, negative relationship.
Prescription opioid deaths	Prescriptions/100 people	2010–2019	10	0.03	0.585	−84	−427	258	No model. Nonsignificant relationship
Opioid treatment admissions/1,000	Prescriptions/100 people	2010–2018	9	0.86	0.000	−32,296	−43,113	−21,478	Strong model. Significant, negative relationship.

## Conclusions

The direct correlations used to justify the CDC guideline and guideline update that existed from 1999 to 2010 are no longer present. Starting in 2010, opioid MME per Capita (POS) does not have a “clear correlation” (7) or move “in parallel” (2) or “in lockstep” (8) with OTA, POD, AOD or TOD. The relationships changed from direct to inverse in 2010. These results hold on a national level, in a large majority of states, and even among patients receiving opioid dosages greater than the recommended maximum dosage in the guideline (much less the reduced maximum dosage recommended in the guideline update). Based on the results presented in this paper and the current trends in opioid deaths, the policies of cutting POS to reduce TOD, AOD, POD, and OTA as presented in the guideline and the guideline update are unfounded and ineffective.

In 2019, the DEA concluded “Without effective new interventions, this overall pattern of predictable exponential growth is likely to continue into the future” (25). Government resources should be allocated to identify the root cause of drug addiction and overdose mortality and then applied to an effective approach that will consistently reduce addiction and overdose deaths.

Reasonable judgment would dictate tracking and reporting of chronic pain patient outcomes (deaths, suicides, returns in benefits, reported pain, function, etc.) for individuals since the guideline or the guideline update. However, there appears to be no publicly available evidence that a monitoring process is required or is planned to measure and confirm outcomes. PDMP records may provide a basis for contact and to survey a random sample of long-term opioid therapy patients to confirm consent, check their status and to evaluate the effectiveness of policy to date.

The results of the analyses presented here help to inform the public, legislators, and the medical community that since 2010 there has been no direct correlation of POS to OTA, POD, AOD, and TOD. The basis for the guideline, the guideline update, communications of public health policy, individual patient care, doctor conduct, congressional testimony, and intergovernmental communication that state and/or imply a direct correlation of POS to OTA, POD, AOD, and TOD are not valid. Based on the current relationships that have existed for a decade the guideline, guideline update and public health policy should be corrected/updated along with an acknowledgment of this material information to avoid misrepresentation or omission of relevant evidence.

## Author contributions

LA obtained all of the data used in the manuscript. BC analyzed the data using regression analysis. Both authors contributed equally to the drafting of the manuscript.

## Conflict of interest

Author BC is employed by Carr Consulting LLC, United States. Author LA is an independent researcher with no current professional affiliations. This study was independent research based on publicly available data. No outside funding was involved in the study design, data acquisition and analysis, decision to publish, or preparation of the manuscript. Both authors declare no other competing interests.

## Publisher's note

All claims expressed in this article are solely those of the authors and do not necessarily represent those of their affiliated organizations, or those of the publisher, the editors and the reviewers. Any product that may be evaluated in this article, or claim that may be made by its manufacturer, is not guaranteed or endorsed by the publisher.
