# Corrigendum: Overdose, opioid treatment admissions and prescription opioid pain reliever relationships: United States, 2010–2019

**DOI:** 10.3389/fpain.2022.1122174

**Published:** 2023-01-20

**Authors:** Editorial Office Frontiers

**Affiliations:** Frontiers Media SA, Lausanne, Switzerland

**Keywords:** prescription opioid sales, overdose deaths, opioid treatment admissions, CDC guideline, statistical analysis, analysis of historical data

A Corrigendum on Overdose, opioid treatment admissions and prescription opioid pain reliever relationships: United States, 2010–2019 By Aubry L and Carr BT. (2022) Front. Pain Res. 3:884674. doi: 10.3389/fpain.2022.884674

**Incorrect Conflict of interest**

In the published article, there was a lack of clarity in the **Conflict of interest** statement. The original statement lacked necessary details and read:

“Author BC was employed by Carr Consulting, United States. The remaining author declares that the research was conducted in the absence of any commercial or financial relationships that could be construed as a potential conflict of interest.”

The correct conflict of interest statement appears below.

**Conflict of interest**

“Author BC is employed by Carr Consulting LLC, United States. Author LA is an independent researcher with no current professional affiliations. This study was independent research based on publicly available data. No outside funding was involved in the study design, data acquisition and analysis, decision to publish, or preparation of the manuscript. Both authors declare no other competing interests.”

The authors apologize for this error and state that this does not change the scientific conclusions of the article in any way. The original article has been updated.

